# A Conversation with Wil Srubar

**DOI:** 10.1021/acscentsci.4c01624

**Published:** 2024-10-04

**Authors:** Robin Donovan

As human populations grow, the built environment expands alongside them, with skyscrapers and multiplexes towering over urban corridors. But manufacturing construction materials usually means burning fossil fuels as we replace green space with concrete.

**Figure:**
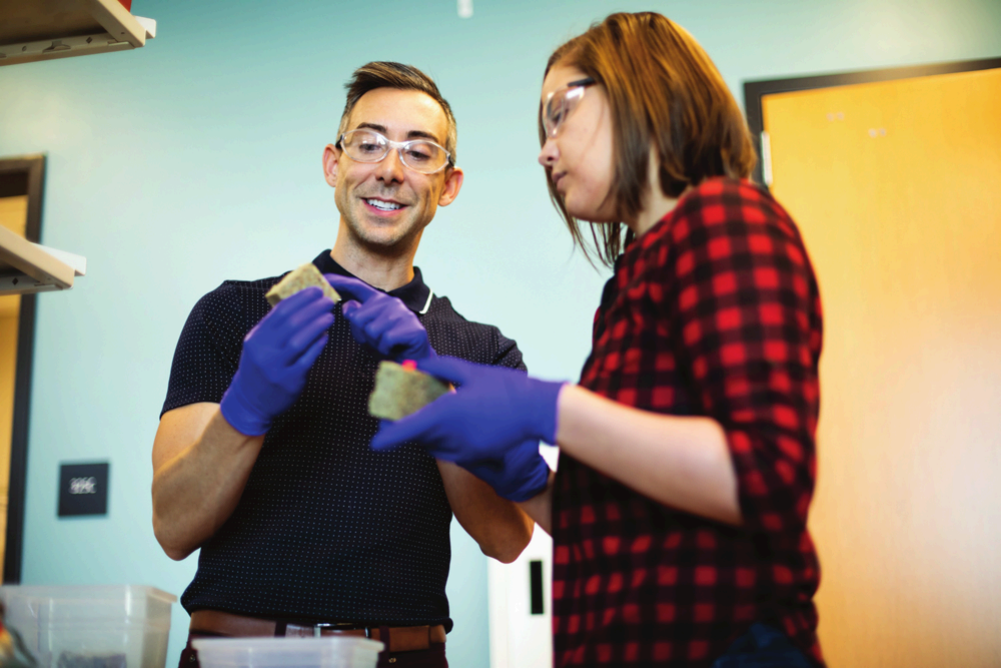
Wil Srubar (left) and then-graduate student Sarah Williams discuss better building materials in the Living Materials Laboratory. Credit: Courtesy of the University of Colorado Boulder.

Wil Srubar hopes to disrupt this cycle with a nature-inspired concrete alternative that can be produced without fossil fuels and massive carbon emissions. It’s just one outcome of work being done in his interdisciplinary Living Materials Laboratory at the University of Colorado Boulder.

A structural engineer, Srubar recruits biologists, chemists, physicists, materials scientists, and a host of engineers to his lab, where they design biomimetic building materials. Think concrete with veinlike systems of stringy fungi that can self-repair cracks, inspired by the human circulatory system; 3D-printed earthen materials reinforced by bacteria and biopolymers; or light-emitting architectural materials designed for use in space that harness natural bioluminescence.

Robin Donovan talked to Srubar about building greener cities, mentorship, and recruiting the next generation of science, technology, engineering, and mathematics (STEM) researchers. This interview was edited for length and clarity.

## What’s the biggest question you’re trying to answer in the Living Materials Laboratory?

How can we blur the boundaries between the built environment and the natural world? The built environment has and will continue to have tremendous environmental consequences. Cement production contributes 8% of [human-driven] global carbon dioxide emissions, and that’s just one material. By learning from nature, harnessing its abilities to produce materials efficiently and sustainably, and blurring the boundaries between the living and the nonliving, we are one step closer to a truly sustainable and regenerative world.

## How is your living concrete different from traditional concrete? Why is it an attractive method for sequestering carbon?

Regular concrete is made by mixing cement powder with water and adding sand, rocks, and other supplementary cementitious materials like fly ash slag, a product of burning coal. Cement is produced by burning limestone, clay, and sometimes other minerals at temperatures up to 1,500 °C. This releases carbon dioxide as limestone decomposes into calcium oxide and carbon dioxide and as we burn fossil fuels to heat the kiln.

The material we invented is chemically different. Instead of burning rocks to make a powder or burning fossil fuels, we rely on ambient temperatures and pressures, as well as certain microorganisms’ innate ability to create minerals that have rocklike properties akin to concrete. At the core of the technology are tiny algae powered by sunlight. These microorganisms produce calcium carbonate, a natural biocement, in a specific biochemical environment. It’s similar to how coral reefs or shells form in the ocean.

Our bioblocks are a concrete alternative that meets and often exceeds the performance specifications set forth by ASTM International for use as a structural and nonstructural concrete masonry unit.

**Figure:**
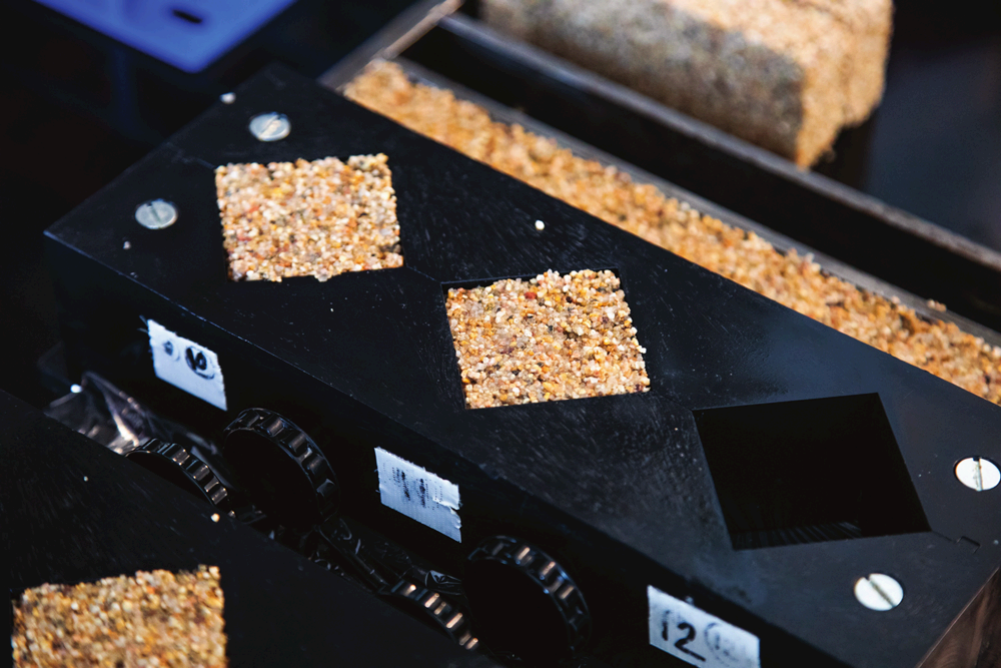
Molds for a brick-like living building material Wil Srubar developed using cyanobacteria. Credit: Courtesy of the University of Colorado Boulder.

## In 20 years, if you’re walking down the street and you’re thinking, Wow, this living concrete was really a slam dunk, what would you see?

I’m an optimist with a realistic perspective. Will we be using nothing but living concrete to build our future cities? My optimistic side says that could be the reality. But I also know that realistically, a myriad of materials would need to be engineered to meet different performance applications.

In the future, I hope to walk down city sidewalks and see living concrete being used in new construction applications, as well as a host of other natural, regenerative materials making up the fabric of not only our built environment but also the products, machines, devices, etc., that we use every day.

Much of the work we do in my lab is to inspire others. The seed we’re planting here is to have other researchers, scientists, and engineers be inspired by what we’re doing and think about other ways we can leverage living organisms to produce materials, structures, and devices.

## You’re hoping to bring your materials to the public through two start-ups and a funding company. What progress have you made, and what is the biggest challenge in translating your work from the lab to urban spaces?

Our dream outcomes are already happening. I cofounded Prometheus Materials, a start-up producing living concrete bioblocks in a facility in Longmont, Colorado.

There has already been an installation in Chicago, where masons built an architectural exhibit with bioblocks as a demonstration project. And the bioblocks have also been used in real buildings, including one in Seattle.

Unlike other industries, construction is always constrained by scale and cost. It’s not that the technology doesn’t work. You have to find methods and processes that are scalable for production and cost-effective to compete with existing materials in target applications.

## Your work isn’t limited to living concrete. What other projects in your lab are you excited about?

My most promising work outside of living concrete relates to carbon-negative cement. We harness the capability of coccolithophores, tiny microalgae, to use sunlight, seawater, and carbon dioxide to grow limestone in real time. This carbon-negative limestone can be used for various building material applications, including the production of carbon-negative cement.

We have also designed and synthesized polymers that mimic the behavior of antifreeze proteins found in nature, making concrete less prone to freeze-thaw damage.

## You’ve won federal funding for recruiting LGBTQ+ students. Why is this an important initiative?

I identify as a first-generation college graduate and a member of the LGBTQ community. Research shows these students are disadvantaged in finding mentors, resources, and support. There’s a retention issue in STEM disciplines.

It’s important to me to be visible and to provide opportunities for these students, something I didn’t have myself. By being an active mentor, I hope folks will see the path to doing the same, be inspired by it, and then choose to pay it forward so we can have an exponential impact. Being visible as an advocate and mentor is so important.

My aspiration is to find follow-on funding once the grant is complete, so we can not only continue the program but also scale it so that other faculty can also provide such opportunities for students.

## We noticed your pup Cooper on your lab’s personnel page. What are his primary responsibilities?

He helps us fetch new ideas! As an undergraduate and graduate student, I didn’t necessarily see the human qualities of my professors. Cooper’s presence reminds everyone that there’s more to life than how good your data are. Plus, he has the best manners.

*Robin Donovan is a freelance contributor to*Chemical & Engineering News, *an independent news publication of the American Chemical Society.*

